# Association between the XRCC1 Polymorphisms and Thyroid Cancer Risk: A Meta-Analysis from Case-Control Studies

**DOI:** 10.1371/journal.pone.0087764

**Published:** 2014-09-11

**Authors:** Fei-Fei Wu, Xiao-Feng He, Hu-Wei Shen, Gui-Jun Qin

**Affiliations:** 1 Department of Endocrinology, First Affiliated Hospital of Zhengzhou University, Zhengzhou, China; 2 Department of Research, Peace Hospital of Changzhi Medical College, Changzhi, China; 3 Department of Endocrinology, Peace Hospital of Changzhi Medical College, Changzhi, China; Universite Libre de Bruxelles (ULB), Belgium

## Abstract

**Background:**

The previous published data on the association between the X-ray repair cross-conplementation group 1 (XRCC1) polymorphisms and thyroid cancer risk remained controversial. Hence, we performed a meta-analysis on all available studies that provided 1729 cases and 3774 controls (from 11 studies) for XRCC1 Arg399Gln, 1040 cases and 2487 controls for Arg194Trp (from 7 studies), and 1432 cases and 3356 controls for Arg280His (from 8 studies).

**Methodology/Principal Findings:**

PubMed, CNKI, and EMBASE database were searched to identify relevant studies. Overall, no significant association was found between XRCC1 Arg399Gln (recessive model: OR = 0.95, 95% CI = 0.77–1.15; dominant model: OR = 0.89, 95% CI = 0.75–1.05; homozygote model: OR = 0.92, 95% CI = 0.69–1.23; Heterozygote model: OR = 0.91, 95% CI = 0.80–1.03; additive model: OR = 0.93, 95% CI = 0.81–1.07), Arg194Trp (recessive model: OR = 1.41, 95% CI = 0.62–3.23; dominant model: OR = 1.01, 95% CI = 0.77–1.34; homozygote model: OR = 1.42, 95% CI = 0.55–3.67; Heterozygote model: OR = 1.03, 95% CI = 0.85–1.26; additive model: OR = 1.08, 95% CI = 0.81–1.42), and Arg280His (recessive model: OR = 1.08, 95% CI = 0.56–2.10; dominant model: OR = 1.01, 95% CI = 0.84–1.22; homozygote model: OR = 1.00, 95% CI = 0.51–1.96; Heterozygote model: OR = 1.04, 95% CI = 0.75–1.42; additive model: OR = 1.03, 95% CI = 0.86–1.23) and thyroid cancer risk when all the eligible studies were pooled into the meta-analysis. In the further stratified and sensitivity analyses, significant association was still not found in these three genetic polymorphisms.

**Conclusions/Significance:**

In summary, this meta-analysis indicates that XRCC1 Arg399Gln, Arg280His, and Arg194Trp are not associated with thyroid cancer.

## Introduction

Thyroid carcinomas are the most frequent endocrine malignancies which among these thyroid carcinomas, more than 90 percent are differentiated thyroid carcinomas (DTC). Pathologically, DTC include papillary, follicular, and Hürthle cell carcinoma [1]. To date, exposure to ionizing radiation is the only known risk factor for thyroid cancer [2]. However, there are evidences that some gene variants including DNA repair genes influence on DTC susceptibility. XRCC1 is one of the candidate genes which its variant relationship with thyroid cancer has not been extensively studied [3].

The *XRCC* (X-Ray cross-complementing) genes were initially discovered through their role in DNA damage response caused by ionizing radiation. They are important components of various DNA repair pathways contributing to DNA-damage processing and genetic stability [4]. X-ray cross-complementing gene 1 (*XRCC1*) is involved in the repair of DNA base damage and singlestrand DNA breaks by binding DNA ligase III at its carboxyl and DNA polymerase β and poly (ADP-ribose) polymerase at the site of damaged DNA [5] and is known to participate in base excision repair (BER) of small lesions such as oxidized or reduced bases, fragmented or nonbulky adducts, and lesions caused by methylating agents [6]. Three common polymorphisms within the *XRCC1* have been identified at codon 194, 280, and 399 (Arg194Trp, Arg280His, and Arg399Gln) [7].

Many studies have reported the association of *XRCC1* polymorphisms at 194, 280, and 399 (Arg194Trp, Arg280His, and Arg399Gln) with thyroid cancer risk [16]–[25], but the results were inconclusive, some original studies thought that these polymorphisms were associated with thyroid cancer risk, but others had different opinions. In addition, attention has been mainly drawn at a meta-analytical level upon the association of *XRCC1* polymorphisms at 194, 280, and 399 with thyroid cancer risk [8], [9]. However, the previous meta-analyses on *XRCC1* Arg194Trp, Arg280His, and Arg399Gln with thyroid cancer risk have shown conflicting conclusions. In order to explore the association between Arg399Gln, Arg194Trp, and Arg280His polymorphisms with thyroid cancer risk, an updated meta-analysis was conducted to summarize the data. Meta-analysis is a good method for summarizing the different studies. It can not only overcome the problem of small size and inadequate statistical power of genetic studies of complex traits, but also provide more reliable results than a single case–control study.

## Materials and Methods

### Identification and eligibility of relevant studies

A bibliographical search was performed in PubMed, CNKI, and EMBASE database to identify studies that evaluated XRCC1 polymorphisms and thyroid cancer up to April 10, 2014. The search terms used were: (polymorphism or mutation or variant) and (XRCC1 or “X-ray repair cross-conplementation group 1”) and thyroid. The search was not limited to language. Additional studies were identified by hand searching references in original articles and review articles. Authors were contacted directly regarding crucial data not reported in original articles. In addition, studies were identified by a manual search of the reference lists of reviews and retrieved studies. We included all the case–control studies and cohort studies that investigated the association between XRCC1 Arg399Gln, Arg194Trp, and Arg280His polymorphisms and thyroid cancer risk with genotyping data. All eligible studies were retrieved, and their bibliographies were checked for other relevant publications. When the same sample was used in several publications, only the most complete information was included following careful examination.

### Inclusion criteria

The included studies needed to have met the following criteria: (1) only the case–control studies or cohort studies were considered, (2) evaluated the XRCC1 Arg399Gln, Arg194Trp, and Arg280His polymorphisms and thyroid cancer risk, and (3) the genotype distribution of the polymorphisms in cases and controls were described in details and the results were expressed as odds ratio (OR) and corresponding 95% confidence interval (95% CI). Major reasons for exclusion of studies were as follows: (1) not for cancer research, (2) only case population, and (3) duplicate of previous publication.

### Data extraction

Information was carefully extracted from all eligible studies independently by two investigators according to the inclusion criteria listed above. The following data were collected from each study: first author's name, year of publication, country of origin, ethnicity, source of controls, genotyping method, and numbers of cases and controls in the XRCC1 Arg399Gln, Arg194Trp, and Arg280His genotypes whenever possible. Ethnicity was categorized as “Caucasian,” “African,” (including African Americans) and “Asian.” We considered the samples of studies from India and Pakistan as of “Indian’” ethnicity, and samples from Middle Eastern countries as “Middle Eastern” ethnicity. When one study did not state which ethnic groups was included or if it was impossible to separate participants according to phenotype, the sample was termed as “mixed population.” We did not define any minimum number of patients to include in this meta-analysis. Articles that reported different ethnic groups and different countries or locations, we considered them different study samples for each category cited above.

### Statistical analysis

Crude odds ratios (ORs) together with their corresponding 95% CIs were used to assess the strength of association between the XRCC1 Arg399Gln, Arg194Trp, and Arg280His polymorphisms and thyroid cancer risk. The pooled ORs were performed for dominant model (Arg399Gln: Arg/Gln+Gln/Gln *versus* Arg/Arg, Arg194Trp: Arg/Trp+Trp/Trp *versus* Arg/Arg, and Arg280His: Arg/Gln+His/His *versus* Arg/Arg); recessive model (Arg399Gln: Gln/Gln *versus* Arg/Gln+Arg/Arg, Arg194Trp: Trp/Trp *versus* Arg/Trp+Arg/Arg, and Arg280His: His/His *versus* Arg/His+Arg/Arg); Homozygote model (Arg399Gln: Gln/Gln *versus* Arg/Arg, Arg194Trp: Trp/Trp *versus* Arg/Arg, and Arg280His: His/His *versus* Arg/Arg), Heterozygote model (Arg399Gln: Arg/Gln *versus* Arg/Arg, Arg194Trp: Arg/Trp *versus* Arg/Arg, and Arg280His: Arg/Gln *versus* Arg/Arg), and additive model (Arg399Gln: Gln *versus* Arg, Arg194Trp: Trp *versus* Arg, and Arg280His: His *versus* Arg), respectively. Heterogeneity assumption was checked by a chi-square-based *Q* test (Heterogeneity was considered statistically significant if *P*<0.10) [26] and quantified using the *I* ^2^ value, a value that describes the percentage of variation across studies that are due to heterogeneity rather than chance, where *I* ^2^ = 0% indicates no observed heterogeneity, with 25% regarded as low, 50% as moderate, and 75% as high [27]. If results were not heterogeneous, the pooled ORs were calculated by the fixed-effect model (we used the *Q*-statistic, which represents the magnitude of heterogeneity between-studies) [28]. Otherwise, a random-effect model was used (when the heterogeneity between-studies were significant) [29]. In addition to the comparison among all subjects, we also performed stratification analyses by ethnicity and histological subtype (papillary thyroid cancer and follicular thyroid cancer). Moreover, the extent to which the combined risk estimate might be affected by individual studies was assessed by consecutively omitting every study from the meta-analysis (leave-one-out sensitivity analysis). This approach would also capture the effect of the oldest or first positive study (first study effect). In addition, sensitivity analysis was also performed, excluding studies whose allele frequencies in controls exhibited significant deviation from the Hardy–Weinberg equilibrium (HWE), given that the deviation may denote bias. Deviation of HWE may reflect methodological problems such as genotyping errors, population stratification or selection bias. HWE was calculated by using the goodness-of-fit test, and deviation was considered when *P*<0.05. Begg's funnel plots [30] and Egger's linear regression test [31] were used to assess publication bias. A meta-regression analysis was carried out to identify the major sources of between-studies variation in the results, using the log of the ORs from each study as dependent variables, and ethnicity and source of controls as the possible sources of heterogeneity. All of the calculations were performed using STATA version 10.0 (STATA Corporation, College Station, TX).

## Results

### Literature Search and Meta-analysis Databases

**Figure 1:**
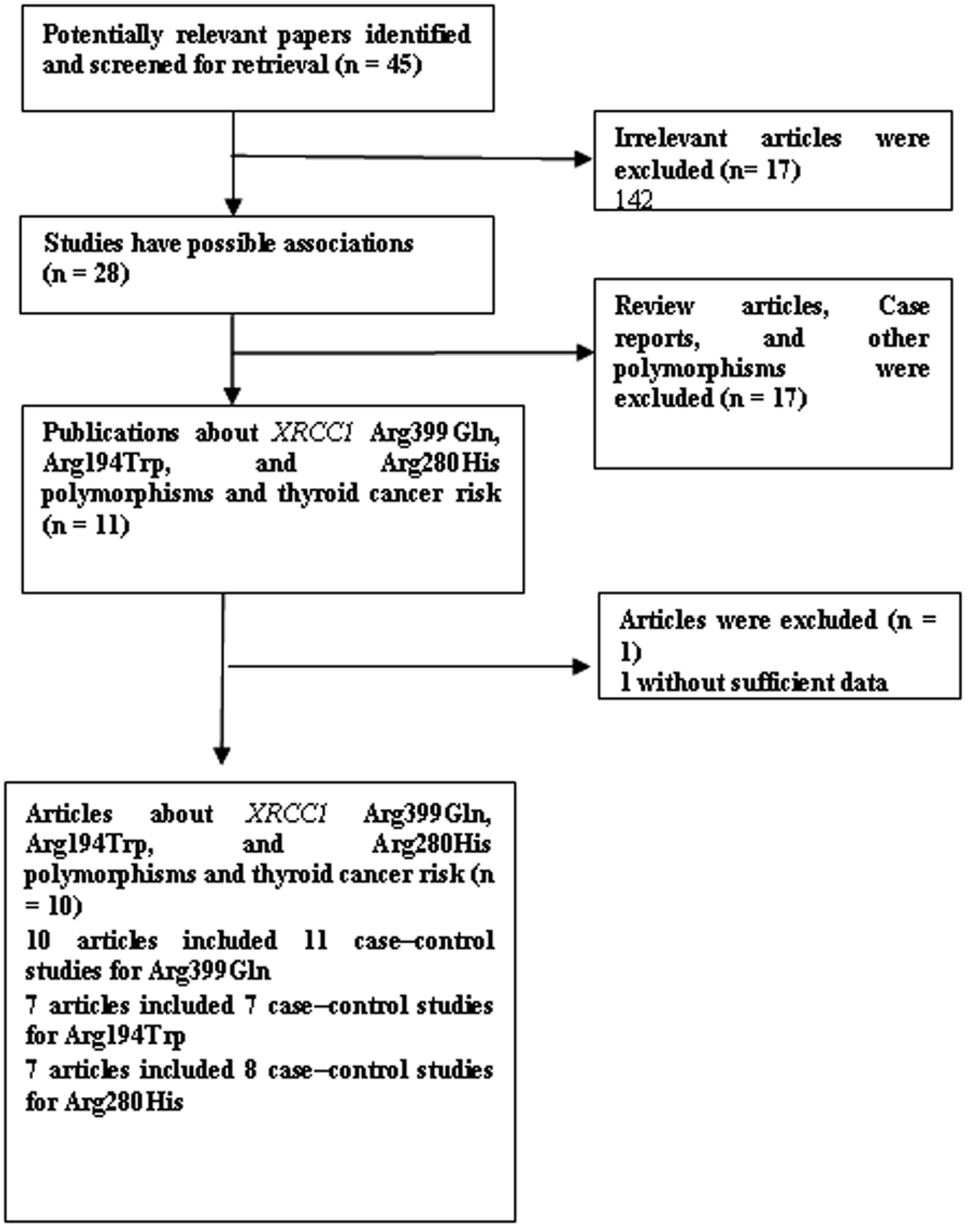
Study flow chart explaining the selection of the 10 eligible articles included in the meta-analysis.

Relevant publications were retrieved and preliminarily screened. As shown in **Fig. 1**, 45 publications were identified, among which 17 irrelevant papers were excluded. Thus, 28 publications were eligible. Among these publications, 17 articles were excluded because they were review articles, case reports, and other polymorphisms of *XRCC1*. In addition, one was excluded because the data of genotyping distribution was missing [32]. As summarized in Table 1, 10 articles with 25 case–control studies publications were selected in the final meta-analysis, including 1729 cases and 3774 controls for *XRCC1* Arg399Gln (from 11 studies), 1,040 cases and 2,487 controls for Arg194Trp (from 7 studies), 1,432 cases and 3,356 controls for Arg280His (from 8 studies). **Tables 1** list all essential information such as the publication year, first author, Country, ethnicity, source of controls, and Genotyping method for XRCC1 Arg399Gln, Arg194Trp, and Arg280His, respectively. Genotype frequencies for thyroid cancer cases and controls were listed in **Table 2** **–** **4**. Among these, two separated case-control studies were included from Akulevich et al. [19] and were considered separately. And one publication was analyzed only in dominant model because Sigurdson et al. [25] provide the limited genotyping information for two XRCC1 polymorphisms (Arg194Trp and Arg280His). Among them, six studies focused on PTC (18, 20, 22, 24, 25) and only Santos et al. [16] on both PTC and FTC. All of the cases were pathologically confirmed.

**Table 1:** Characteristics of studies included in the meta-analysis.

First author	Year	Country	Ethnicity	SC	SNP	Genotyping method
Santos [16]	2012	Portugal	Caucasian	HB	Arg399Gln	PCR-RFLP
Santos [16]	2012	Portugal	Caucasian	HB	Arg194Trp	PCR-RFLP
Fard-Esfahani [17]	2011	Iran	Caucasian	HB	Arg399Gln	PCR-RFLP
Fard-Esfahani [17]	2011	Iran	Caucasian	HB	Arg194Trp	PCR-RFLP
Fard-Esfahani [17]	2011	Iran	Caucasian	HB	Arg280His	PCR-RFLP
Ryu [18]	2011	Korea	Asian	HB	Arg399Gln	PCR-RFLP
Ryu [18]	2011	Korea	Asian	HB	Arg194Trp	PCR-RFLP
García-Quispes [19]	2011	Spain	Caucasian	HB	Arg399Gln	iPLEX
García-Quispes [19]	2011	Spain	Caucasian	HB	Arg280His	iPLEX
Akulevich [20]	2009	RB	Caucasian	HB	Arg399Gln	PCR-RFLP
Akulevich [20]	2009	RB	Caucasian	PB	Arg399Gln	PCR-RFLP
Akulevich [20]	2009	RB	Caucasian	HB	Arg280His	PCR-RFLP
Akulevich [20]	2009	RB	Caucasian	PB	Arg280His	PCR-RFLP
Ho [21]	2009	USA	Mixed	HB	Arg399Gln	PCR-RFLP
Ho [21]	2009	USA	Mixed	HB	Arg194Trp	PCR-RFLP
Ho [21]	2009	USA	Mixed	HB	Arg280His	PCR-RFLP
Siraj [22]	2009	Saudi	ME	HB	Arg399Gln	PCR-RFLP
Siraj [22]	2009	Saudi	ME	HB	Arg280His	PCR-RFLP
Chiang [23]	2008	China	Asian	HB	Arg399Gln	Taqman
Chiang [23]	2008	China	Asian	HB	Arg194Trp	Taqman
Chiang [23]	2008	China	Asian	HB	Arg280His	Taqman
Zhu [24]	2004	China	Asian	HB	Arg399Gln	PCR-RFLP
Zhu [24]	2004	China	Asian	HB	Arg194Trp	PCR-RFLP
Sigurdson [25]	2009	Kazakhstan	Mixed	N	Arg399Gln	Taqman
Sigurdson [25]	2009	Kazakhstan	Mixed	N	Arg194Trp	Taqman
Sigurdson [25]	2009	Kazakhstan	Mixed	N	Arg280His	Taqman

HT, Histological type; RB Russia and Belarus, HB hospital-based studies, N nested case-control studies, PB population-based studies, SC source of controls,

**Table 2:** Genotype distribution of XRCC1 Arg399Gln polymorphism used in the meta-analysis.

First author	Year	Case			Control			HWE	MAF
		Arg/Arg	Arg/Gln	Gln/Gln	Arg/Arg	Arg/Gln	Gln/Gln		
Santos [16]	2012	46	50	13	87	105	25	0.43	0.36
Fard-Esfahani [17]	2011	78	60	17	83	87	20	0.69	0.33
Ryu [18]	2011	87	17	7	72	19	9	<0.01	0.19
García-Quispes [19]	2011	153	186	47	196	212	66	0.48	0.36
Akulevich [20]	2009	65	53	14	158	193	47	0.30	0.36
Akulevich [20]	2009	55	50	18	75	100	22	0.18	0.37
Ho [21]	2009	133	99	19	220	216	67	0.23	0.35
Siraj [22]	2008	35	13	2	142	72	15	0.16	0.22
Chiang [23]	2008	150	110	23	277	165	27	0.71	0.23
Zhu [24]	2004	49	44	12	57	45	3	0.09	0.24
Sigurdson [25]	2009	12	10	2	460	343	89	0.036	0.29

HWE, Hardy-Weinberg equilibrium; MAF minor aller freqyency; Arg, the major allele, Gln, the minor allele.

**Table 3:** Genotype distribution of XRCC1 Arg280His polymorphism used in the meta-analysis.

First author	Year	Case			Control			HWE	MAF
		Arg/Arg	Arg/His	His/His	Arg/Arg	Arg/His	His/His		
Fard-Esfahani [17]	2011	146	23	1	173	18	2	0.07	0.06
García-Quispes [19]	2011	337	58	3	426	44	3	0.12	0.05
Akulevich [20]	2009	117	15	0	366	32	0	0.40	0.04
Akulevich [20]	2009	113	10	0	176	19	0	0.47	0.05
Ho [21]	2009	229	22	0	453	50	0	0.24	0.05
Siraj [22]	2009	33	12	5	129	79	21	0.09	0.26
Chiang [23]	2008	224	54	5	349	113	7	0.53	0.14
Sigurdson [25]	2009	24	1		800	96		-	-

HWE, Hardy-Weinberg equilibrium; MAF minor aller freqyency; Arg, the major allele, His, the minor allele.

**Table 4:** Genotype distribution of XRCC1 Arg194Trp polymorphism used in the meta-analysis.

First author	Year	Case			Control			HWE	MAF
		Arg/Arg	Arg/Trp	Trp/Trp	Arg/Arg	Arg/Trp	Trp/Trp		
Santos [16]	2012	98	8	2	196	21	0	0.45	0.05
Fard-Esfahani [17]	2011	136	18	3	166	20	1	0.64	0.06
Ryu [18]	2011	59	43	9	37	49	14	0.73	0.39
Ho [21]	2009	203	45	3	433	69	1	0.31	0.07
Zhu [24]	2004	50	52	3	48	51	6	0.11	0.30
Sigurdson [25]	2009	20	5		665	241		-	-
Chiang [23]	2008	127	119	37	234	199	36	0.48	0.29

HWE, Hardy-Weinberg equilibrium; MAF minor aller freqyency; Arg, the major allele; Trp, the minor allele.

### Quantitative synthesis

**Table 5** listed the main results of the meta-analysis of *XRCC1* polymorphisms and thyroid cancer risk. For Arg399Gln, there was no significant association between this polymorphism and thyroid cancer risk in any genetic model when all the eligible studies were pooled together. Similarly, the combined results did not showed any association between Arg194Trp/Arg280His polymorphisms and thyroid cancer risk for all genetic models. However, in the subgroup analysis by ethnicity, the results showed that Arg/His genotype was associated with an increased risk of thyroid cancer among Caucasians (dominant model: OR = 1.43, 95% CI = 1.08–1.89, *P* value of heterogeneity test [*P*_h_] = 0.513, *I* ^2^ = 0.0%; additive model: OR = 1.38, 95% CI = 1.05–1.80, *P*_h_ = 0.551, *I* ^2^ = 0.0%; Heterozygote model: OR = 1.45, 95% CI = 1.09–1.93, *P*_h_ = 0.495, *I* ^2^ = 0.0%). And carriers of the 399Gln variant allele have a decreased thyroid cancer risk in mixed population (dominant model: OR = 0.73, 95% CI = 0.55–0.97, *P*_h_ = 0.326, *I* ^2^ = 0.0%; additive model: OR = 0.73, 95% CI = 0.59–0.92, *P*_h_ = 0.308, *I* ^2^ = 3.6%; recessive model: OR = 0.56, 95% CI = 0.34–0.93, *P*_h_ = 0.588, *I* ^2^ = 0.0%; homozygote model: OR = 0.50, 95% CI = 0.30–0.85, *P*_h_ = 0.460, *I* ^2^ = 0.0%). We also detected that the Trp allele of Arg194Trp polymorphism was significantly increased thyroid cancer risk in mixed population (additive model: OR = 1.49, 95% CI = 1.02–2.17). When subgroup analysis by histological subtype, the results showed that Arg194Trp polymorphism was associated with decreased papillary thyroid cancer (PTC) risk in dominant model (OR = 0.71, 95% CI = 0.50–0.99, *P*_h_ = 0.525, *I* ^2^ = 0.0%).

**Table 5:** Results of meta-analysis for Arg399Gln, Arg194Trp, and Arg280His polymorphisms and the risk of thyroid cancer.

Generic model		Recessive model			Dominant model			Homozygote			Heterozygote			Additive model		
Arg399Gln	n	Gln/Gln vs. Arg/Gln+Arg/Arg			Arg/Gln+Gln/Gln vs. Arg/Arg			Gln/Gln vs. Arg/Arg			Arg/Gln vs. Arg/Arg			Gln vs. Arg		
		OR (95%CI)	*P_h_*	*I^2^* (%)	OR (95%CI)	*P_h_*	*I^2^* (%)	OR (95%CI)	*P_h_*	*I^2^* (%)	OR (95%CI)	*P_h_*	*I^2^* (%)	OR (95%CI)	*P_h_*	*I^2^* (%)
Overall	11 (1729/3774)	0.95 (0.77–1.15)	0.160	30.0	0.89 (0.75–1.05)	0.089	39.1	0.92 (0.69–1.23)*	0.090	38.9	0.91 (0.80–1.03)	0.240	21.4	0.93 (0.81–1.07)*	0.031	49.5
Ethnicity																
Caucasian	5 (905/1476)	0.97 (0.75–1.26)	0.822	0.0	0.87 (0.74–1.03)	0.342	11.2	0.91 (0.69–1.19)	0.937	0.0%	0.87 (0.72–1.04)	0.193	34.2	0.92 (0.82–1.05)	0.744	0.0
Asian	3 (499/674)	1.50 (0.64–3.50)	0.086	59.2	1.19 (0.93–1.51)	0.217	34.6	1.56 (0.63–3.86)*	0.067	63.1	1.14 (0.88–1.47)	0.451	0.0	1.15 (0.81–1.63)*	0.086	59.2
Mixed	2 (275/1395)	**0.56 (0.34**–**0.93)**	0.588	0.0	**0.73 (0.55**–**0.97)**	0.326	0.0	**0.50 (0.30**–**0.85)**	0.460	0.0%	0.80 (0.59–1.07)	0.403	0.0	**0.73 (0.59**–**0.92)**	0.308	3.6
Histological subtype																
PTC	7 (623/2138)	1.13 (0.82–1.57)	0.323	14.1	0.85 (0.70–1.04)	0.382	5.9	1.02 (0.73–1.43)	0.238	25.1	0.82 (0.66–1.01)	0.620	0.0	0.94 (0.80–1.09)	0.188	31.4

Arg194Trp	n	Trp/Trp vs. Arg/Trp+Arg/Arg			Arg/Trp+Trp/Trp vs. Arg/Arg			Trp/Trp vs. Arg/Arg			Arg/Trp vs. Arg/Arg			Trp vs. Arg		
		OR (95%CI)	*P_h_*	*I^2^* (%)	OR (95%CI)	*P_h_*	*I^2^* (%)	OR (95%CI)	*P_h_*	*I^2^* (%)	OR (95%CI)	*P_h_*	*I^2^* (%)	OR (95%CI)	*P_h_*	*I^2^* (%)
Overall	7 (1040/2487)	1.41 (0.62–3.23)	0.041	56.8	1.01 (0.77–1.34)	0.088	45.6	1.42 (0.55–3.67)*	0.014	65.1	1.03 (0.85–1.26)	0.202	31.2	1.08 (0.81–1.42)*	0.019	63.1
Ethnicity																
Caucasian	2 (265/404)	5.38 (0.89–32.5)	0.591	0.0	1.10 (0.67–1.82)	0.634	0.0	5.37 (0.89–32.5)	0.605	0.0	0.95 (0.56–1.60)	0.509	0.0	1.25 (0.79–1.99)	0.779	0.0
Asian	3 (499/674)	0.88 (0.33–2.32)	0.025	72.9	0.87 (0.52–1.44)*	0.026	72.7	0.78 (0.24–2.52)*	0.007	79.8	0.95 (0.74–1.21)	0.118	53.2	0.90 (0.57–1.43)*	0.006	80.7%
Mixed	2 (276/1409)	6.07 (0.63–58.7)	–	–	1.30 (0.90–1.87)	0.168	47.4	6.40 (0.66–61.9)	–	–	1.39 (0.92–2.10)	–	–	**1.49 (1.02–2.17)**	–	–
Histological subtype																
PTC	4 (318/1328)	0.71 (0.35–1.40)	0.112	54.3	**0.71 (0.50–0.99)**	0.525	0.0	0.75 (0.18–3.12)*	0.088	58.8	0.71 (0.49–1.03)	0.314	13.8	0.76 (0.58–1.01)	0.302	16.5

Arg280His	n	His/His vs. Arg/His+Arg/Arg			Arg/His+His/His vs. Arg/Arg			His/His vs. Arg/Arg			Arg/His vs. Arg/Arg			His vs. Arg		
		OR (95%CI)	*P_h_*	*I^2^* (%)	OR (95%CI)	*P_h_*	*I^2^* (%)	OR (95%CI)	*P_h_*	*I^2^* (%)	OR (95%CI)	*P_h_*	*I^2^* (%)	OR (95%CI)	*P_h_*	*I^2^* (%)
Overall	8 (1432/3356)	1.08 (0.56–2.10)	0.957	0.0	1.01 (0.84–1.22)*	0.061	48.2	1.00 (0.51–1.96)	0.958	0.0	1.04 (0.75–1.42)*	0.035	55.9	1.03 (0.86–1.23)	0.118	40.9
Ethnicity																
Caucasian	4 (823/3356)	0.94 (0.25–3.50)	0.614	0.0	**1.43 (1.08**–**1.89)**	0.513	0.0	0.99 (0.26–3.70)	0.608	0.0	**1.45 (1.09**–**1.93)**	0.495	0.0	**1.38 (1.05**–**1.80)**	0.551	0.0
Mixed	2 (276/1399)	–	–	–	0.80 (0.48–1.32)	0.382	0.0	–	–	–	0.87 (0.51–1.47)	–	–	0.88 (0.52–1.46)	–	–
Histological subtype																
PTC	4 (330/1718)	1.10 (0.39–3.07)	–	–	0.87 (0.59–1.29)	0.263	24.7	0.93 (0.33–2.65)	–	–	0.91 (0.61–1.38)	0.173	43.0	0.95 (0.67–1.36)	0.316	13.2

1 All summary ORs were calculated using fixed-effects models. In the case of significant heterogeneity (indicated by *), ORs were calculated using random-effects models.

### Test of heterogeneity and sensitivity

There was significant heterogeneity among these studies for dominant model comparison (Arg399Gln: *P*_h_ = 0.089, Arg194Trp: *P*_h_ = 0.088, and Arg280His: *P*_h_ = 0.061), recessive model (Arg194Trp: *P*_h_ = 0.041), homozygote model comparison (Arg399Gln: *P*_h_ = 0.090, Arg194Trp: *P*_h_ = 0.014), heterozygote model (Arg280His: *P*_h_ = 0.035), and additive model comparison (Arg399Gln: *P*_h_ = 0.031, Arg194Trp: *P*_h_ = 0.019). Then, we assessed the source of heterogeneity by ethnicity and source of controls. The results of meta-regression indicated that ethnicity (dominant model: *P* = 0.039 for Arg399Gln and *P* = 0.001 for Arg280His; additive model: *P* = 0.001 for Arg399Gln; homozygote model: *P* = 0.002 for Arg399Gln; heterozygote model: *P*<0.001 for Arg280His) but not source of controls (dominant model: *P* = 0.799 for Arg399Gln and *P* = 0.086 for Arg280His; additive model: *P* = 0.500 for Arg399Gln; homozygote model: *P* = 0.388 for Arg399Gln; heterozygote model: *P* = 0.159 for Arg280His) contributed to substantial heterogeneity among the meta-analysis. Although there were two studies [18], [25] deviated from HWE for Arg399Gln polymorphism, the corresponding pooled ORs were not materially altered by excluding these studies in overall and subgroup analyses. However, when the study of Ho et al. [21] was excluded, the results were changed in mixed population for Arg399Gln (dominant model: OR = 1.06, 95% CI = 0.47–2.40; additive model: OR = 1.00, 95% CI = 0.53–1.88; recessive model: OR = 0.82, 95% CI = 0.19–3.55; homozygote model: OR = 0.86, 95% CI = 0.19–3.92).

For Arg194Trp polymorphism, when one study was excluded, the results were also changed in mixed population (data not shown) and PTC (dominant model: OR = 0.85, 95% CI = 0.55–1.29). For Arg280His polymorphism, when one study was excluded, the results were also changed in Caucasians (dominant model: OR = 1.25, 95% CI = 0.84–1.85; additive model: OR = 1.21, 95% CI = 0.83–1.76; Heterozygote model: OR = 1.28, 95% CI = 0.86–1.90).

### Publication bias

**Figure 2:**
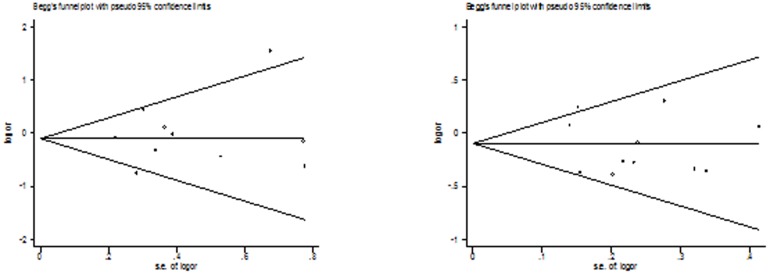
Begg's funnel plot of the meta-analysis of thyroid cancer risk and XRCC1 Arg399Gln polymorphism. (Homozygote model and dominant model).

Both Begg's funnel plot and Egger's test were performed to access the publication bias of this meta-analysis. Begg's funnel plots did not reveal any evidence of obvious asymmetry in any genetic model in the overall meta-analysis (**Figure 2** **–** **4**). The Egger's test results also suggested no evidence of publication bias in the meta-analysis of Arg399Gln (*P* = 0.523 for dominant model, *P* = 0.466 for recessive model, *P* = 0.796 for additive model, *P* = 0.598 for homozygote model, and *P* = 0.329 for heterozygote model), Arg194Trp (*P* = 0.224 for dominant model, *P* = 0.758 for recessive model, *P* = 0.618 for additive model, *P* = 0.822 for homozygote model, and *P* = 0.293 for heterozygote model), and Arg280His (*P* = 0.656 for dominant model, *P* = 0.236 for recessive model, *P* = 0.821 for additive model, *P* = 0.588 for homozygote model, and *P* = 0.992 for heterozygote model), respectively.

**Figure 3:**
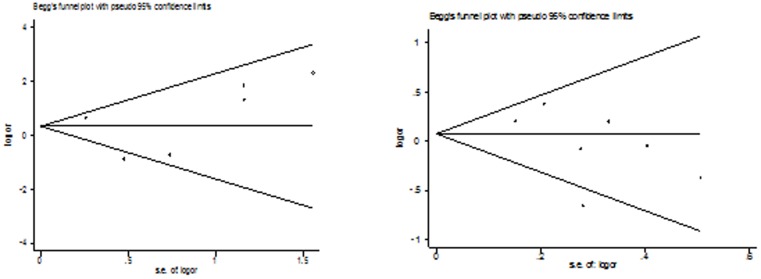
Begg's funnel plot of the meta-analysis of thyroid cancer risk and XRCC1 Arg194Trp polymorphism. (Homozygote model and dominant model).

**Figure 4:**
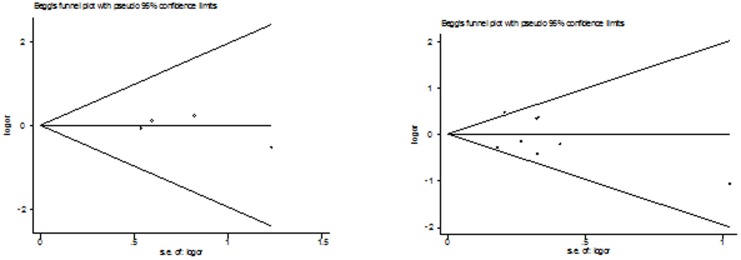
Begg's funnel plot of the meta-analysis of thyroid cancer risk and XRCC1 Arg194Trp polymorphism. (Homozygote model and dominant model).

## Discussion

DNA is continuously damaged by endogenous and exogenous mutagens and carcinogens. The damages are fixed by multiple DNA repair pathways including base excision repair, nucleotide excision repair, mismatch repair, and double-strand break repair [15]. Cells with unrepaired DNA damage undergo either apoptosis or unregulated growth to malignancy. A defect or reduced efficiency in repairing DNA damage therefore plays a pivotal role in the development of cancer. One of the DNA repair genes exhibiting polymorphic variation is XRCC1, which is located on chromosome 19q13.2 and encodes a M_r_ 70,000 protein [10]. XRCC1 (X-ray cross-complementing group 1 protein) is involved in the repair of DNA base damage and single-strand DNA breaks by binding DNA ligase III at its carboxyl and DNA polymerase β and poly (ADP-ribose) polymerase at the site of damaged DNA [11]. Deletion of the XRCC1 gene in mice results in an embryonic lethal phenotype [12]. Chinese hamster ovary cell lines with mutations in the XRCC1 have shown a reduced ability to repair single-strand breaks in DNA and concomitant cellular hypersensitivity to ionizing radiation and alkylating agents [13]. These suggest that XRCC1 plays an essential role in the removal of endogenous and exogenous DNA damage. Three polymorphisms in coding regions of the XRCC1 gene at codons 194 (Arg to Trp), 280 (Arg to His), and 399 (Arg to Gln) have been recently identified [14]. A number of epidemiological studies have evaluated the association between XRCC1 Arg399Gln, Arg194Trp, and Arg280His polymorphisms and thyroid cancer risk, but the results remain inconclusive. In order to resolve this conflict, a meta-analysis was performed to examine the association between *XRCC1* polymorphisms and thyroid cancer risk, by critically reviewing 11 studies on *XRCC1* Arg399Gln (a total of 1729 cases and 3774 controls), 7 studies on Arg194Trp (1040 cases and 2487 controls), and 8 studies on Arg280His (1432 cases and 3356 controls).

Overall, no significant association was found between *XRCC1* Arg399Gln, Arg280His, and Arg194Trp when all the eligible studies were pooled into the meta-analysis. And In the further stratified and sensitivity analyses, significant association was still not found in these three genetic polymorphisms. Zhu et al. [24] in 2004, Santos et al. [16], Sigurdson et al. [25], and Ho et al. [21] reported that the XRCC1 Arg194Trp was not associated with the risk of thyroid cancer. Ryu et al. [18] in 2011, Santos et al. [16], Sigurdson et al. [25], García-Quispes et al. [19], Fard-Esfahani et al. [17], Chiang et al. [23] and Akulevich et al. [20] reported that the XRCC1 Arg399Gln polymorphism was not associated with the risk of thyroid cancer. Sigurdson et al. [25], Fard-Esfahani et al. [17] Akulevich et al. [20], and Chiang [23] et al. reported that the XRCC1 Arg280His polymorphism was not associated with the risk of thyroid cancer. The results of our meta-analysis supported the negative association between XRCC1 Arg399Gln, Arg194Trp, and Arg280His polymorphisms and thyroid cancer risk. However, a careful matching should be considered in future larger genetic association studies including multiple ethnic groups. In the present meta-analysis, between-studies heterogeneity was observed for XRCC1 Arg399Gln, Arg280His, and Arg194Trp. The results of meta-regression indicated that ethnicity but not source of controls contributed to substantial heterogeneity among the meta-analysis of Arg280His and Arg399Gln. Hence, the same polymorphisms may play different roles in different ethnicity, because cancer is a complicated multi-genetic disease, and different genetic backgrounds may contribute to the discrepancy. And even more importantly, the low penetrance genetic effects of single polymorphism may largely depend on interaction with other polymorphisms and/or a particular environmental exposure.

Previous meta-analyses on *XRCC1* Arg399Gln, Arg194Trp, and Arg280His polymorphisms with thyroid cancer risk showed conflicting results. The study of Hu et al. [33] suggested that XRCC1 Arg399Gln polymorphism is not associated with differentiated thyroid carcinoma risk, while a decreased risk is observed among Caucasian population. The study of Qian et al. [8] suggested that XRCC1 Arg399Gln polymorphism might be associated with decreased thyroid cancer risk among Caucasians and XRCC1 Arg194Trp may be associated with a tendency for increased thyroid cancer risk in the two larger sample size trials. The study of Bao et al. [9] suggested that Arg280His might contribute to the susceptibility of Differentiated Thyroid Carcinoma (DTC) among Caucasians, whereas it might provide protective effects in Asians against the risk of DTC. Additionally, their results supported the protective role of Arg194Trp polymorphism in developing PTC, and showed evidence of an association between Arg399Gln polymorphism and decreased risk of DTC in mixed population. The study of Du et al. [34] suggested that XRCC1 Arg194Trp may be a risk factor for DTC development. The study of Wang et al. [35] demonstrated that the XRCC1 Arg399Gln, Arg194Trp, and Arg280His may be associated with developing of thyroid cancer. However, the results of the present meta-analysis are not in accordance with those reported by the previous meta-analysis [8], [9], [33]–[35]. Our meta-analysis indicates that XRCC1 Arg399Gln, Arg280His, and Arg194Trp are not associated with thyroid cancer. Our results seem to confirm and establish the trend in the meta-analysis of the XRCC1 Arg399Gln, Arg280His, and Arg194Trp polymorphisms because this meta-analysis performed a more complete sensitivity analysis than the previous meta-analysis [8], [9], [33]–[35]. We found that previous meta-analysis [8], [9], [33]–[35] did not seriously perform the sensitivity analysis. hence, their meta-analysis results may be inaccurate.

There are several limitations in this meta-analysis. First, the controls were not uniformly defined. Although most of them were common populations, some controls were population-based; other controls were hospital-based. Hence, non–differential misclassification bias is possible. Second, in the subgroup analysis may have had insufficient statistical power to check an association, Third, we were also unable to examine the interactions among gene-environment, lacking of the original data of the included studies limited our further evaluation of potential interactions, which may be an important component of the association between XRCC1 Arg399Gln, Arg280His, and Arg194Trp polymorphisms and environment and thyroid cancer risk. Four, it was much difficult to get the all articles published in various language. Last, our results were based on unadjusted published estimates. Because of data limitations, we were unable to adjust them such as age and alcohol consumption et al. Our meta-analysis also has several strengths. First, a meta-analysis of the association of XRCC1 Arg399Gln, Arg280His, and Arg194Trp polymorphisms with thyroid cancer risk is statistically more powerful than any single study. Second, the quality of eligible studies included in current meta-analysis was satisfactory and met our inclusion criterion.

In summary, this meta-analysis indicates that XRCC1 Arg399Gln, Arg280His, and Arg194Trp are not associated with thyroid cancer. However, it is necessary to conduct large sample studies using standardized unbiased genotyping methods, homogeneous cancer patients and well-matched controls. Moreover, further studies estimating the effect of gene–gene and gene–environment interactions may eventually lead to our better, comprehensive understanding of the association between the XRCC1 Arg399Gln, Arg280His, and Arg194Trp polymorphisms and thyroid cancer risk.

## Supporting Information

Checklist S1 **PRISMA Checklist.**
(DOC)
